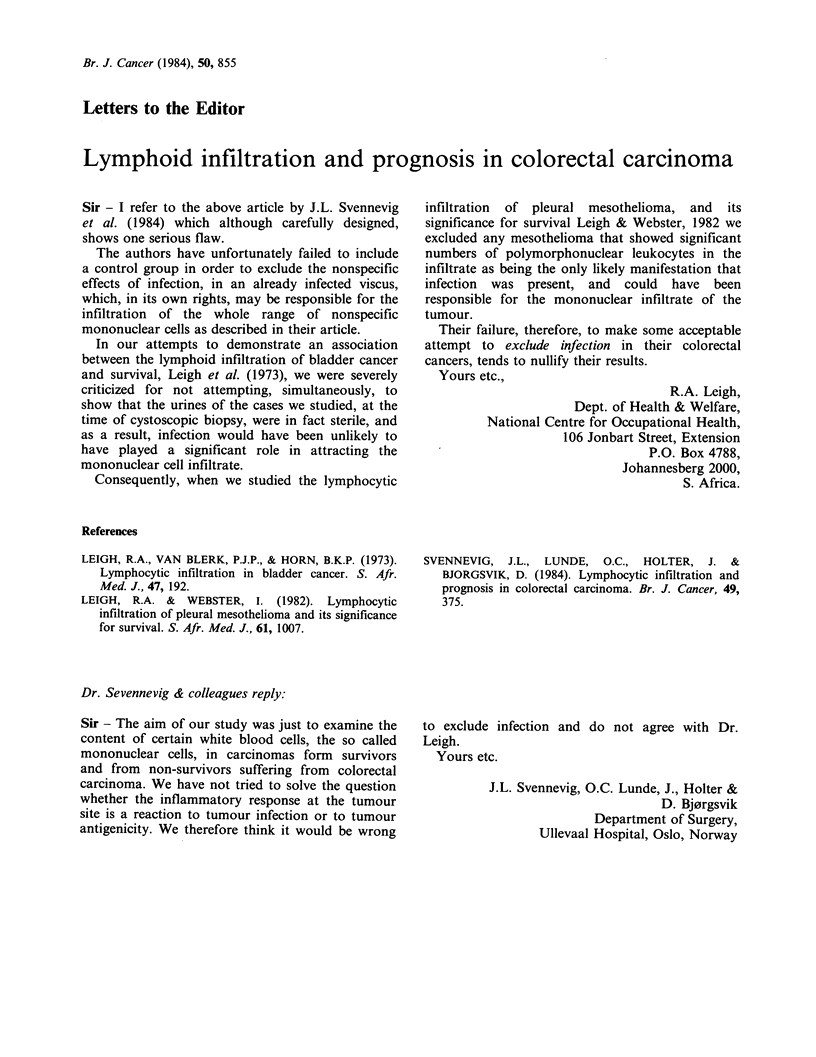# Lymphoid infiltration and prognosis in colorectal carcinoma

**Published:** 1984-12

**Authors:** J.L. Svennevig, O.C. Lunde, J. Holter, D. Bjørgsvik


*Dr. Sevennevig & colleagues reply:*

**Sir** – The aim of our study was just to examine the content of certain white blood cells, the so called mononuclear cells, in carcinomas form survivors and from non-survivors suffering from colorectal carcinoma. We have not tried to solve the question whether the inflammatory response at the tumour site is a reaction to tumour infection or to tumour antigenicity. We therefore think it would be wrong to exclude infection and do not agree with Dr. Leigh.

Yours etc.

J.L. Svennevig, O.C. Lunde, J., Holter &
D. Bjørgsvik
Department of Surgery,
Ullevaal Hospital, Oslo, Norway